# Association between arsenic exposure and plasma cholinesterase activity: a population based study in Bangladesh

**DOI:** 10.1186/1476-069X-9-36

**Published:** 2010-07-10

**Authors:** Nurshad Ali, Md Ashraful Hoque, Abedul Haque, Kazi Abdus Salam, Md Rezaul Karim, Aminur Rahman, Khairul Islam, Zahangir Alam Saud, Md Abdul Khalek, Anwarul Azim Akhand, Mostaque Hossain, Abul Mandal, Md Rezaul Karim, Hideki Miyataka, Seiichiro Himeno, Khaled Hossain

**Affiliations:** 1 Department of Biochemistry and Molecular Biology, Rajshahi University, Rajshahi-6205, Bangladesh; 2 Department of Statistics, Rajshahi University, Rajshahi-6205, Bangladesh; 3 Department of Genetic Engineering and Biotechnology, Dhaka University, Dhaka-1000, Bangladesh; 4 Department of Medicine, Rajshahi Medical College Hospital, Rajshahi-6000, Bangladesh; 5 School of Life Sciences, University of Skövde, PO Box 408, SE-541 28 Skövde, Sweden; 6 Department of Applied Nutrition and Food Technology, Islamic University, Kushtia-7003, Bangladesh; 7 Laboratory of Molecular Nutrition and Toxicology, Faculty of Pharmaceutical Sciences, Tokushima Bunri University, Tokushima 770-8514, Japan

## Abstract

**Background:**

Arsenic is a potent pollutant that has caused an environmental catastrophe in certain parts of the world including Bangladesh where millions of people are presently at risk due to drinking water contaminated by arsenic. Chronic arsenic exposure has been scientifically shown as a cause for liver damage, cancers, neurological disorders and several other ailments. The relationship between plasma cholinesterase (PChE) activity and arsenic exposure has not yet been clearly documented. However, decreased PChE activity has been found in patients suffering liver dysfunction, heart attack, cancer metastasis and neurotoxicity. Therefore, in this study, we evaluated the PChE activity in individuals exposed to arsenic via drinking water in Bangladesh.

**Methods:**

A total of 141 Bangladeshi residents living in arsenic endemic areas with the mean arsenic exposure of 14.10 ± 3.27 years were selected as study subjects and split into tertile groups based on three water arsenic concentrations: low (< 129 μg/L), medium (130-264 μg/L) and high (> 265 μg/L). Study subjects were further sub-divided into two groups (≤50 μg/L and > 50 μg/L) based on the recommended upper limit of water arsenic concentration (50 μg/L) in Bangladesh. Blood samples were collected from the study subjects by venipuncture and arsenic concentrations in drinking water, hair and nail samples were measured by Inductively Coupled Plasma Mass Spectroscopy (ICP-MS). PChE activity was assayed by spectrophotometer.

**Results:**

Arsenic concentrations in hair and nails were positively correlated with the arsenic levels in drinking water. Significant decreases in PChE activity were observed with increasing concentrations of arsenic in water, hair and nails. The average levels of PChE activity in low, medium and high arsenic exposure groups were also significantly different between each group. Lower levels of PChE activity were also observed in the > 50 μg/L group compared to the ≤50 μg/L group. Moreover, PChE activity was significantly decreased in the skin (+) symptoms group compared to those without (-).

**Conclusions:**

We found a significant inverse relationship between arsenic exposure and PChE activity in a human population in Bangladesh. This research demonstrates a novel exposure-response relationship between arsenic and PChE activity which may explain one of the biological mechanisms through which arsenic exerts its neuro-and hepatotoxicity in humans.

## Background

Arsenic is a ubiquitous element present in food, soil, water and airborne particles. The general population is exposed to inorganic and organic arsenic through water, food, occupation and other environmental sources. It has been reported that a large section of the population in Bangladesh and in West Bengal, India have been exposed to arsenic through arsenic contaminated drinking water [1-3]. Bangladesh has already experienced the largest, global catastrophe due to the presence of excessive amounts of arsenic in drinking water [3]. A significant number of toxicity cases have been already reported in the north-west region in Bangladesh and approximately 16 million additional people are currently at risk for arsenic toxicity in the country. The situation is deteriorating as the new cases of arsenic poisoning are still being reported in different parts of the country. Although arsenic is a well established human carcinogen, paradoxically, arsenic is also used to treat acute promyelocytic leukemia (APL) due to its capacity to induce apoptosis in cancer cells [4]. Due to its controversial medical status as both a therapeutic treatment and as an acute toxigen, there are renewed interests in exploring its potential benefits and disadvantages. Arsenic in drinking water is typically inorganic, and can be present either as As^+3 ^(arsenite) or As^+5 ^(arsenate). In Bangladesh, arsenic in ground water is primarily in the As^+3 ^form which is more toxic than the pentavalent (As^+5^) form [5]. As^+3 ^has higher affinity to thiol groups of proteins and enzymes compared to the As^+5 ^[6]. Most of the adverse effects of arsenic are caused by aberrant intracellular signalling pathways whereby arsenic reacts with the thiol groups of proteins and enzymes and changes their catalytic activity.

Arsenic toxicity induces dermatitis, multi-site cancers, cardiovascular diseases, diabetes mellitus, immune disorders, peripheral neuropathy, liver damage, renal failure, and other illnesses [2,7-12]. Hepatic disorders including hepatic cancer appear to be a primary cause of arsenic-related mortality [13-15]. Previously Guha Mazumder [16] conducted a study in an Indian population exposed to arsenic and showed that prolonged drinking of arsenic contaminated water was associated with hepatomegaly characterized by increased activity of hepatic enzymes that are used for liver function test. Oxidative DNA damage, acquired tolerance to apoptosis, enhanced cell proliferation, altered DNA methylation, genomic instability and aberrant estrogen signaling [17,18] have been reported in patients with liver toxicity caused by arsenic.

Neuropathy and neurological disorders are observed in cases of both acute and chronic arsenic toxicity [19]. The clinical features of neuropathy are paresthesias, numbness and pain, particularly in the soles of the feet. Arsenic-induced neuropathy specifically is revealed by a reduced nerve conduction velocity. Most of the adverse effects of arsenic on nervous system are caused by inactivated enzymes in the cellular energy pathway, whereby arsenic reacts with the thiol groups of proteins and enzymes and inhibits their catalytic activity. Furthermore, arsenic-induced neurotoxicity, like many other neurodegenerative diseases, causes changes in cytoskeletal protein composition and hyperphosphorylation. These changes may lead to disorganization of the cytoskeletal framework, which is a potential mechanism of arsenic-induced neurotoxicity.

True cholinesterase or acetyl cholinesterase is highly specific for the neurotransmitter acetylcholine and catalyzes the hydrolysis of acetylcholine into choline and acetic acid, a reaction necessary to allow a cholinergic neuron to return to its resting state after activation. Cholinesterase is a member of the serine hydrolase family, which utilizes a serine residue at the active site [20]. Humans have three types of cholinesterase: red blood cell (RBC) cholinesterase, called "true cholinesterase," plasma cholinesterase, called "pseudo cholinesterase," and brain cholinesterase. Acetylcholine causes the stimulation of neurons while cholinesterase causes the ending of stimulation by breaking down the acetylcholine. Cholinesterase inhibiting chemicals cause breakdown of this balance, leading to the development of neurotoxicity. Red blood cell cholinesterase is the same enzyme that is found in the nervous system, while the plasma cholinesterase also known as butyryl cholinesterase is made in the liver. Pseudo cholinesterase (PChE) has a broader range of esterase activity that can hydrolyze butyryl choline, acetylcholine and other aliphatic esters. Cholinesterase activity was reported to be decreased in heart attack, liver dysfunction, cancer metastasis [21], muscular tremors, and neurological disorders. Decreased cholinesterase activity is used to monitor the toxicity of some pesticides such as organophosphate and carbamates that are associated with the development of liver diseases, peripheral neuropathy, neuropsychiatric abnormalites and extra pyramidal disorders [21-27].

Although PChE activity is used to monitor the liver dysfunction and neurotxicity in humans exposed to several toxic chemicals, the enzymatic activity has not yet been evaluated clearly in the population chronically exposed to arsenic. In this study, we therefore, assayed the PChE activity in the human subjects exposed to arsenic to evaluate the relationship between arsenic exposure and PChE activity.

## Methods

### Study areas

The selected study areas were chosen according to the arsenic affected area map http://www.dchtrust.org/arsenic_map.htm presented by the School of Environmental Studies (SOES), Jadavpur University, India and Dhaka Community Hospital, Bangladesh. Preliminary information about the arseniasis-endemic areas was also collected from the local health office. The study area included Marua in Jessore, Dutpatila and Vultie in Chuadanga and Bheramara in Kushtia district (north-west region) of Bangladesh. Prevalence of typical skin symptoms of arsenicosis such as melanosis on the skin, hyperkeratosis and hard patches on the palms and soles were very high in the local residents of these areas.

### Ethical permission

Ethical permission for this study was approved by the Bangladesh Medical Research Council, Mohakhali, Dhaka-1212 (Ethical clearance no. BMRC/ERC/2007-2010/558). All sorts of confidentialities and rights of the study subjects were strictly maintained as per the guideline of the Bangladesh Medical Research Council.

### Water collection and arsenic analysis

The drinking-water was entirely supplied by the tube wells which were set up by the government or by individuals. The tube well water was used for drinking, cooking and other household purposes. We selected only those tube wells that were being used by the local residents for their drinking water. Water samples from these tube wells were collected in acid-washed containers after the well was pumped for five minutes as previously described [28]. Total arsenic concentration in water samples was determined by Inductively Coupled Plasma Mass Spectroscopy (ICP-MS, HP-4500, Agilent Technologies, Kanagawa, Japan) after the addition of a solution of yttrium (10 ppb in 1.0% nitric acid) to all water samples as an internal standard for ICP-MS analysis. The ion signals for arsenic and yttrium were monitored at m/z of 75 and 79, respectively. All samples were determined in triplicate and the average values were used for data analysis. The detection limit of As^75 ^was 30 ppt. CRM, "river water" (NMIJ CRM 7202-a No.347 National Institute of Advanced Industrial Science and Technology, Japan) was used as a certified reference material. The average value (mean ± SD) of arsenic in the "river water" determined in triplicate by ICP-MS analysis was 1.06 ± 0.04 μg/L (reference value, 1.18 μg/L).

### Study subjects

Sequentially, drinking water sources (tube well water) in the study areas and then study subjects who were drinking water from those sources were selected. The study subjects were selected without knowing their arsenic exposure level in drinking water. The water from one tube well is used either by the members of one family or by the members of the several families. We recruited our study subjects based on the largest possible number of family members or families who used water from the selected tube wells. Elderly persons (> 70 years of age), pregnant women, lactating mothers, children (< 12 years of age) and patients who underwent recent surgical operation were excluded from this study. The individuals, who had previous and recent histories of drug addiction, hepatitis, hepatoxic drugs, malaria, and kalazar, and farmers exposed to any kind of agricultural/home insecticides within last one month, were also excluded as these could influence PChE activity. Of the 153 individuals who were approached, 141 were recruited (92.16% participation rate) and 12 were excluded. We selected the study subjects irrespective of symptoms; however, subjects with skin symptoms were identified by a clinical doctor first and then were further confirmed by a dermatologist. We found that multiple members in one family suffered from typical visible symptoms of arsenicosis such as melanosis on the skin and hyperkeratosis and hard patches on the palms and soles. The doctor involved in this study carefully checked many parts of the body for the confirmation of the melanosis and hyperkeratosis.

### Personal household interview

The subjects participated in this study gave their written consent. Household visits were carried out to interview residents. Personal interview of the study subjects was carried out by our research team using a standard questionnaire. Information obtained from the interview included the sources of water for drinking and daily house hold uses, water consumption history, socioeconomic status, occupation, food habit, cigarette smoking, personal and family medical history, history of diseases, physiological complications, major diseases, previous physicians reports, Body Mass Index (BMI), recent history of agricultural and home insecticides or pesticides exposure, uses of air fresheners, aerosol, mosquito coils, and consumption of alcohol and tari (locally fermented coconut palms).

### Collection of nail and hair samples, and analysis of arsenic poisoning

Arsenic levels in nail and hair samples have been reported to provide the integrated measures for arsenic exposure [29,30]. Nail samples were collected from each study subject as described previously [31]. Hair samples with the length of about 1 cm were collected from the region of the head close to the scalp behind the ear by using a ceramic blade cutter and kept in polypropylene bottles [32]. Nail and hair samples were cleaned by the method described by Chen et al. [33]. Briefly, samples were immersed in 1% Triton X-100, sonicated for 20 minutes, and then washed five times with milli-Q water. The washed samples were allowed to dry at 60°C overnight in a drying oven. Nail and hair samples were digested with concentrated nitric acid using a hot plate at 70°C for 15 minutes and 115°C for 15 minutes. After cooling, the samples were diluted with 1.0% nitric acid containing yttrium (10 ppb), and concentrations of As^75 ^and Y^79 ^in these samples were determined by ICP-MS. Determination of the accurate arsenic concentrations in these biological materials was verified by using a CRM "cod fish powder" (NMIJ CRM 7402-a, National Institute of Advanced Industrial Science and Technology, Japan). The average value (mean ± SD) of arsenic in "cod fish powder" determined in triplicate by the above-mentioned digestion followed by ICP-MS analysis was 34.9 ± 2.35 μg/g (reference value, 36.7 μg/g).

### Collection of blood plasma

All study subjects were requested to gather in a convenient nearby locality. Fasting blood samples were collected from the study subjects. Blood samples (5-7 ml) were collected from each individual by venipuncture in the EDTA-containing blood collection tubes. Whole blood was then placed immediately on ice and subsequently centrifuged at 1,600 × g for 15 minutes at 4°C. Plasma supernatant was then taken and stored at -80°C.

### Laboratory examination

PChE activity was measured using butyryl cholinesterase (CHE) kit according to the manufacture's protocol (RANDOX, UK). The principle of colorimetric method is briefly described below: Butyryl cholinesterase hydrolyses butyrylthiocholine to give thiocholine and butyrate. The reaction between thiocholine and dithiobis (nitrobenzoate) gives 2-nitro-5-mercaptobenzoate, a yellow compound which can be measured at 405 nm (Microlab 200, Vital Scientific, Dieren, Netherlands). All human samples were analyzed in triplicate, and then mean values were taken.

### Statistical analysis

Statistical analysis for this study was performed by using software of Statistical Packages for Social Sciences (SPSS). The study subjects were split into tertile groups based on drinking water arsenic concentrations of equal proportions based on frequency testing: low (< 129 μg/L), medium (130-264 μg/L) and high (> 265 μg/L). We performed Spearman correlation coefficient test to evaluate the correlation of water arsenic with hair or nail arsenic in low, medium and high exposure groups; the correlation of water arsenic concentrations with hair or nail arsenic concentrations and to evaluate the association between PChE activity and the concentration of arsenic in water or hair or nails. PChE activity in low, medium and high exposure groups was evaluated by one way ANOVA. PChE activity between the groups based on the regulatory upper limit of arsenic concentration in drinking water in Bangladesh and on the prevalence of skin symptoms were analyzed by Independent Samples T-Test. Effects of age, sex, arsenic concentration in water and BMI were analyzed by main effect model (four-way classification).

## Results

### General characteristics of the study subjects

Table 1 shows the characteristics of the study subjects in the low, medium and high exposure groups depending on the water arsenic concentrations. There were total 52 female and 89 male subjects with a mean age of 37.66 ± 12.51 years. Most of the female study subjects were house wives (90.70%) and the remaining 9.30% were farm workers, tailors or other professions. Most of the male subjects were farmers (85.54%) and remaining 14.46% were small vendors, rickshaw puller or other professions. The average duration of chronic arsenic exposure for all study subjects was 14.10 ± 3.27 years. The mean concentrations of water arsenic in the low, medium and high groups were 32.13 ± 43.55, 215.82 ± 36.96 and 426.80 ± 91.10 μg/L, respectively. It has been confirmed by questionnaire that the study subjects were not exposed to any kind of agricultural/home insecticides or pesticides at least for the last month. The mean BMI of the low, medium and high arsenic exposure groups were within normal range: 20.89 ± 3.24, 20.32 ± 3.08 and 20.11 ± 3.35, respectively. Approximately 70% of the study subjects showed typical symptoms of arsenicosis such as diffused or spotted melanosis or hyperkeratosis on the skin, foot and palms.

**Table 1:** General characteristics of the study subjects based on water arsenic

Characteristics	All Subjects	Low (< 129 μg/L)	Medium (130 - 264 μg/L)	High (> 265 μg/L)
No. of subject	141	44	50	47
Sex [no. (male/female)]	89/52	32/12	32/18	25/22
Age [years (Mean ± SD)]	37.66 ± 12.51	39.34 ± 13.71	35.22 ± 10.93	38.43 ± 12.90
Water arsenic concentration [μg/L (Mean ± SD)]	224.92 ± 57.20	32.13 ± 43.55	215.82 ± 36.96	426.80 ± 91.10
Water consumption (L/day)	2.30 ± 1.09	1.71 ± 1.01	2.54 ± 1.13	2.65 ± 1.15
Years of arsenic exposure (Mean ± SD)	14.10 ± 3.27	16.00 ± 3.10	13.20 ± 3.20	13.10 ± 3.50
Occupation				
Female	90.70	87.34	90.21	94.54
Housewives (%)	9.30	12.66	9.79	5.46
Others (%) [Farmworkers, tailors etc.]				
Male				
Farmers (%)	85.54	85.26	87.45	83.91
Others (%) [Venders, rickshaw pullers etc.]	14.46	14.74	12.55	16.09
(+) symptomps [% (Melanosis and hyperkeratosis)]	69.52	61.37	60	87.2
(-) symptomps (%)	30.48	38.63	40	12.8
BMI*	20.44 ± 3.22	20.89 ± 3.24	20.32 ± 3.08	20.11 ± 3.35

BMI* was calculated as body weight (kg) divided by square body height (m^2^)

Frequency test is used to divide the study subjects into three groups of equal proportion based on water arsenic concentration

### Hair and nail arsenic concentrations in low, medium and high exposure groups

Table 2 shows the hair and nail arsenic concentrations in low (< 129 μg/L), medium (130-264 μg/L) and high (> 265 μg/L) arsenic exposure groups. Arsenic concentrations in water showed significant positive correlations with arsenic concentrations in hair and nails in each of three groups and also in all subjects.

**Table 2:** Hair & nail arsenic concentrations and their correlations with water arsenic concentrations

Water arsenic exposure	n	Hair arsenic concentration		Nail arsenic concentration	
		[μg/g (Mean ± SD)]	Spearman *r*	[μg/g (Mean ± SD)]	Spearman *r*
Low (< 129)	44	1.83 ± 3.00	0.413**	3.51 ± 4.41	0.421**
Medium (130-264)	50	4.08 ± 2.60	0.489**	6.32 ± 5.62	0.384*
High (> 265)	47	9.74 ± 10.13	0.464**	12.51 ± 9.13	0.499***
All subjects	141	5.27 ± 7.06	0.524***	7.51 ± 7.64	0.541***

Here *p < 0.05, **p < 0.01 and ***p < 0.001 are from Spearman correlation coefficient test

### Correlations of water arsenic concentrations to arsenic concentrations in hair and nails

**Figure 1:**
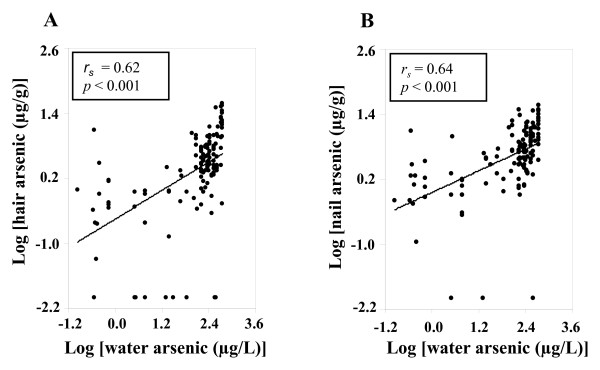
**Dose-related relationship of water arsenic with hair and nail arsenic concentrations**. Relationship between drinking water arsenic and the arsenic in hair (A) or in nails (B) of the study subjects. Arsenic concentrations in water, hair and nails were used after log transformation. *r_s _*and *p*- values are from Spearman correlation coefficient test.

Water arsenic concentrations showed positive dose-dependent relationship (*r*_s _= 0.62, *p *< 0.001) with hair arsenic concentrations (Figure 1A). Similarly, a positive relationship (*r*_s _= 0.64, *p *< 0.001) was observed between water and nail arsenic concentrations (Figure 1B).

### PChE activity and arsenic exposure

**Figure 2:**
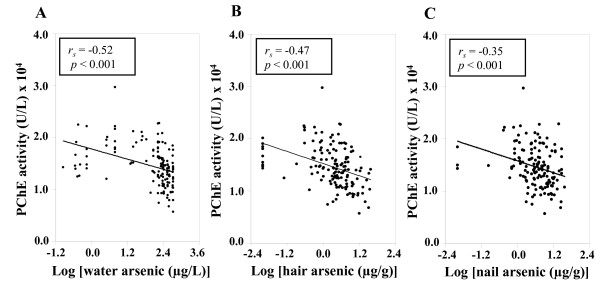
**Effect of arsenic exposure on PChE activity**. Effects of drinking water (A), hair (B) and nail (C) arsenic concentrations on PChE activity. Arsenic concentrations were used after log transformation. *r_s _*and *p*- values are from Spearman correlation coefficient test.

**Figure 3:**
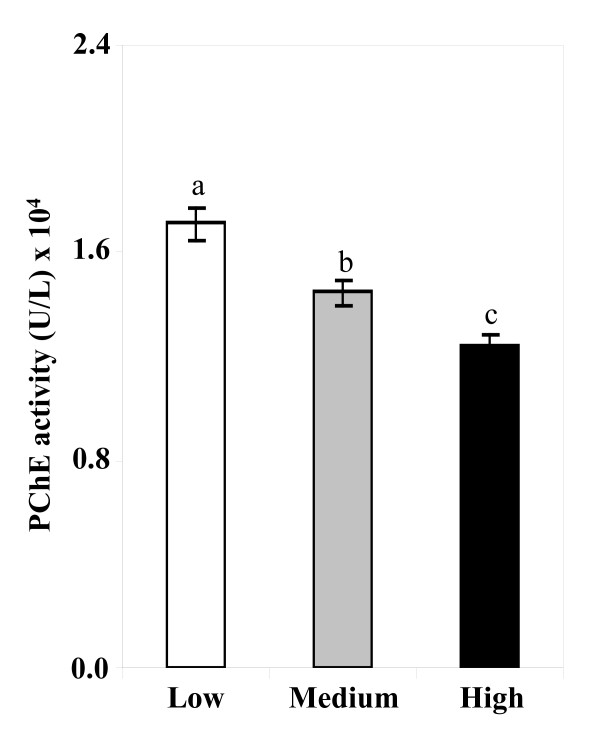
**PChE activity of the low, medium and high exposure group of arsenic**. Blank, gray and black bars represent the PChE activity (Mean ± SE) of low (n = 44), medium (n = 50) and high (n = 47) exposure groups of study subjects respectively. *p-*values are from one way ANOVA test. ^a ^Significantly different from medium group at *p *< 0.01. ^b ^Significantly different from high group at *p *< 0.01. ^c ^Significantly different from low group at *p *< 0.001.

Figure 2 shows the effect of arsenic exposure on PChE activity. A significant decrease in PChE activity was observed with the increasing concentrations of arsenic in drinking water (Figure 2A) with a significant negative correlation (*r*_s _= -0.52, *p *< 0.001). A similar relationship was observed between PChE activity and hair arsenic concentrations (*r*_s _= -0.47, *p *< 0.001, Figure 2B), and between PChE activity and nail arsenic concentrations (*r*_s _= -0.35, *p *< 0.001, Figure 2C). The level of PChE activity was found to be significantly decreased in medium versus low (*p *< 0.01), high versus medium (*p *< 0.01) and high versus low (*p *< 0.001) arsenic exposure groups (Figure 3). Furthermore, we divided the study subjects into two groups (Table 3) based on the recommended upper limit (50 μg/L) of arsenic concentration in water for Bangladesh, and found that PChE activity was significantly (*p <*0.001) lower in the > 50 μg/L group than in the ≤50 μg/L group. Usually skin symptoms such as melanosis on the skin, hyperkeratosis and hard patches on the palms and soles are developed as a result of prolonged exposure of arsenic. The majority of our study subjects (approximately 70%) had typical skin symptoms of arsenicosis. Therefore, we next compared the PChE activity based on the presence of skin symptoms (Table 3). We categorized the study subjects into two groups of with (+) and without (-) symptoms. Intriguingly, PChE activity was significantly decreased in the skin (+) symptoms group compared to the without (-) symptoms group.

**Table 3:** PChE activity in the groups based on the skin symptoms and on the regulatory upper limit of arsenic concentration (50 μg/L) in drinking water

Parameters	Categories	n	PChE activity (U/L) × 10^4 ^(Mean ± SD)	*p*-value
Skin symptoms	(-) Symptoms	43	1.693 ± 0.394	0.000
	(+) Symptoms	98	1.359 ± 0.349	
Arsenic in drinking water	≤ 50 μg/L	33	1.775 ± 0.371	0.000
	> 50 μg/L	108	1.365 ± 0.349	

*p*-values are from Independent Samples T-Test

### Effects of age, sex, water arsenic and BMI on PChE activity

We checked the effects of confounders (age, sex, water arsenic concentration and BMI) on PChE activity. Results from the main effect model (four-way classification) showed that there were no significant effects of age, sex and BMI on PChE activity (Table 4). On the other hand, a clear significant association (*p *< 0.001) between PChE activity and water arsenic concentrations was observed. The main effect model controlled for age, sex, water arsenic levels and BMI.

**Table 4:** Effects of age, sex, water arsenic and BMI on PChE activity

Parameters	SS	df	Mean Square	F
Age	2011860.248	2	1005930.124	0.079
Sex	6020517.227	1	6020517.227	0.471
Water arsenic	335859312.541	2	167929656.270	13.13*
BMI	41162709.416	2	20581354.708	1.609
Total	32276437312			

Effects are from four-way classification (main effect model). *p < 0.001

## Discussion

In this study, we assayed the PChE activity in the population of the severely arsenic-contaminated areas in the north-west region of Bangladesh. The decreased activity of cholinesterase was reported previously in rats that were injected with high doses of arsenic only for five days [34]. The arsenic-mediated decrease in cholinesterase activity in an animal model prompted us to examine the status of this enzyme in a human population that was exposed to arsenic. We found that hair and nail arsenic concentrations were positively correlated with the arsenic concentrations in drinking water (Figure 1). These results were in agreement with the previous report [35], which suggested that arsenic content of hair and nail samples might be used as an effective biomarker of arsenic intake. PChE activity in this population was decreased with increasing levels of arsenic in the drinking water (Figure 2A). Similar effects were also observed with the increasing levels of arsenic in hair and nails (Figure 2B and 2C, respectively). Consistent results across these three exposure metrics explicitly stated the potential role of chronic arsenic exposure in lowering PChE activity in humans. We further examined the PChE activity splitting the study subjects into tertile groups based on water arsenic levels. Interestingly, a lower level of PChE activity was observed in higher tertile groups (Figure 3). Since a previous study indicated that males were more vulnerable to arsenic toxicity than females [36], we examined the effects of confounding factors such as age, sex and BMI on PChE activity. However, no significant effect of these factors was detected (Table 4). Therefore, all these results provided important evidence to conclude that arsenic exposure could be an important contributor for lowering PChE activity in humans.

While this manuscript was under preparation, Hernandez's et al. [37] reported that the activity of cholinesterase was not decreased by arsenic. The reasons for this discrepancy may be attributable to the differences in study population and levels of arsenic exposure between the two studies. Hernandez's group did not examine the effect of arsenic in drinking water. They selected healthy population from Spain who had much lower levels of arsenic in urine. Whereas, in our study subjects, the exposures occurred for much longer periods of time and were in much higher levels resulting in a visible dermal toxicity in the majority (approximately 70%) of the population. Thus, it is possible that the arsenic-mediated decrease in PChE activity may be limited to a population who are chronically exposed to high level of arsenic from contaminated drinking water. This notion is supported by a report of an animal model experiment that showed an inverse relationship between high level of arsenic and cholinesterase activity [34]. Further studies are needed to examine whether the PChE activity decreases or not at lower exposures for prolonged periods of time.

Decreased PChE activity has also been reported earlier to be associated with the exposure of the pesticides such as organophosphate and carbamates [22]. To avoid the involvement of confounding effects such as exposure to insecticides or pesticides, we confirmed, prior to the sample collection, that our study subjects did not use any kind of agricultural/home insecticides or pesticides for no less than the past month. Many of our study subjects for the most part were uneducated about the general dangers related to exposure to home insecticides and/or pesticides. This population belonging to Bangladesh's low socio-economic group were unfamiliar with the use of mosquito coils, aerosol, air fresheners, etc. in their daily lives for protection. Prior to sample collection, we explained to them about some home insecticides and asked them to provide us with information regarding their past and present exposure to these types of insecticides. In addition, we performed an alternative experiment selecting some subjects (n = 22) from the city (non-agricultural region) where the possibilities of drinking water pollution by pesticides or insecticides were very low. The levels of arsenic in the drinking water for these subjects had not crossed the maximum permissive limit and accordingly, the hair and nail arsenic levels were also low. We observed that the PChE activity of this group was almost the same compared to that of the lower arsenic exposure group of the study subject in arseniasis-endemic areas (unpublished results). These results maintain that the possible role of confounders (pesticides/insecticides) on PChE activity in this study might be very low or negligible. Nevertheless, we can not exclude the possibility that the effects of some chemicals are involved in the decrease in PChE activity in our study subjects. However, if another accompanying chemical agent in drinking water could also be responsible for the observed association between arsenic and PChE activity, the chemical would follow the same concentration gradient as arsenic in the drinking water. This is unlikely, but more detailed examination of the chemical components in drinking water may be required in a future study.

The mechanisms for how arsenic decreases cholinesterase activity have not yet been documented. Arsenic compounds are known as potent inhibitor of many enzymes [38] and the reaction between the arsenic and the free sulfhydryl groups of enzymes may underlie the mechanism of this inhibition. However, cholinesterase does not have the structural features suitable for binding with arsenic. Cholinesterase has been found to contain cysteine only in the form of disulphide bridges and not as free thiol [39]. Moreover, other reactant chemicals in the sulfhydryl group such as iodoacetamide or mercury compounds did not inhibit cholinesterase [40]. Therefore, arsenic reduces the activity of cholinesterase probably due to other mechanisms rather than the direct binding to the enzyme.

Cholinesterase is considered to be a clinically important enzyme because it is involved in both liver function abnormalities and neurotoxicity by several toxic chemicals. Despite the deleterious action of arsenic on liver and central nervous system, there have been very limited studies of the effect of arsenic on liver and nervous system. Therefore, the present study provided new insights into liver function abnormalities and neurotoxicity caused by arsenic. We have also a future plan to conduct a follow up study to investigate the level of PChE activity in our study subjects. Many of them have already obtained opportunities for drinking relatively safe water containing reduced levels of arsenic provided by the several government and non-governmental organizations in the arseniasis-endemic areas in Bangladesh. Reduced levels of arsenic concentration in water are expected to increase the PChE activity to its normal level and decrease the risk of arsenic-induced pathogenesis.

## Conclusions

In this study, we found that hair and nail arsenic concentrations were positively correlated with arsenic concentrations in drinking water in a human population exposed to arsenic in Bangladesh. A significant inverse relationship between arsenic exposure and PChE activity was observed. Thus, this research demonstrates a novel exposure-response relationship between arsenic and PChE activity which may explain one of the biological mechanisms through which arsenic exerts its neuro-and hepatotoxicity in humans.

## Abbreviations

ICP-MS: Inductively Coupled Plasma Mass Spectroscopy; PChE: Plasma cholinesterase (butyryl cholinesterase); APL: Acute promyelocytic leukemia.

## Competing interests

The authors declare that they have no competing interests.

## Authors' contributions

NA and MAH were responsible for specimen collection, data analysis, summarization of results and manuscript preparation. AH, KAS, MRK, AR, KI, ZAS, MAK were taken part in data collection, processing and analysis. AAA was involved in study design and interpretation of results. MH was involved in study design, characterization of study subjects and data collection. AM was involved in the study design and preparation of manuscript. MRK gave general support in specimen collection, interpretation of results and manuscript preparation. HM and SH carried out the analysis of arsenic exposure by estimating arsenic in water, hair and nails. KH took the overall responsibility in hypothesis generation, study design, specimen collection, data analysis, and manuscript preparation. All authors read and approved the final manuscript.
